# Effects of Quality Enhancement of Frozen Tuna Fillets Using Ultrasound-Assisted Salting: Physicochemical Properties, Histology, and Proteomics

**DOI:** 10.3390/foods13040525

**Published:** 2024-02-08

**Authors:** Yuke He, Zhou Zhao, Yaogang Wu, Zhiyuan Lu, Caibo Zhao, Juan Xiao, Zhiqiang Guo

**Affiliations:** 1 Hainan Engineering Research Center of Aquatic Resources Efficient Utilization in South China Sea, Key Laboratory of Seafood Processing of Haikou, Key Laboratory of Food Nutrition and Functional Food of Hainan Province, School of Food Science and Engineering, Hainan University, Haikou 570228, China; 21210832000009@hainanu.edu.cn (Y.H.);; 2 School of Marine Science and Engineering, Hainan University, Haikou 570228, China

**Keywords:** ultrasound, salting, 4D label-free proteomics, yellowfin tuna (*Thunnus albacares*), quality, frozen storage

## Abstract

Salting pretreatment is an effective method to improve the quality of frozen fish. This study investigated the quality changes and proteomic profile differences of frozen yellowfin tuna fillets pretreated with ultrasound-assisted salting (UAS) and static salting (SS). This study was centered on three aspects: physicochemical indicators’ determination, histological observation, and proteomic analysis. The results showed that UAS significantly increased yield, salt content, and water-holding capacity (WHC), decreased total volatile base nitrogen (TVBN) compared to SS (*p* < 0.05), and significantly increased water in the protein matrix within myofibrils. Histological observations showed that the tissue cells in the UAS group were less affected by frozen damage, with a more swollen structure and rougher surface of myofibrils observed. Furthermore, 4D label-free proteomics revealed 56 differentially abundant proteins (DAPs) in UAS vs. NT comparison, mainly structural proteins, metabolic enzymes, proteasomes, and their subunits, which are associated with metabolic pathways such as calcium signaling pathway, gap junction, actin cytoskeletal regulation, and necroptosis, which are intimately associated with quality changes in freeze-stored tuna fillets. In brief, UAS enhances the potential for the application of salting pretreatment to improve frozen meat quality, and 4D label-free proteomics provides knowledge to reveal the potential links between quality and molecular changes in processed frozen meat to optimize future UAS meat processing.

## 1. Introduction

Yellowfin tuna is a marine fish with high economic value, with a total global catch of more than 1.5 million tons in 2021 [1]. After being caught in distant waters, tuna is usually rapidly frozen at low temperatures and then sold mainly as whole fish or fillets [2,3]. Cryogenic freezing delays its spoilage and deterioration, but the formation of ice crystals during freezing inevitably causes mechanical damage to its tissues, and thus, the damaged tissues are more susceptible to the effects of the surrounding environment (such as low temperatures, ice crystal formation, etc.) [2,4]. Then, under the influence of endogenous enzymes and bacteria, the fish tissues will undergo adverse changes, such as the loss of juices, color browning, and structural breakdown [5]. To reduce the negative impacts on frozen products, several pretreatments before freezing have been considered to improve the quality of frozen meat, such as the modification of meat properties or the addition of cryoprotectants.

Low-concentration salting (about 1 mol/L NaCl) not only results in tender and succulent fish but also effectively inhibits yield loss and quality deterioration caused by freezing [6,7]. It has been reported that the proper salting pretreatment can improve the water-holding capacity (WHC) and texture stability of thawed meat by regulating the morphology of ice crystals in frozen meat and the recoverability of thawed tissues [2]. However, traditional salting methods rely on localized concentration gradients, which are time-consuming and ineffective [2,3]. Several studies have combined salting with various methods to shorten the salting time, including ultrasound [8], vacuum impregnation and injection [9], etc. Currently, ultrasound technology has been applied to assist in the salting process of meats to improve salting efficiency and improve quality characteristics, such as beef, pork, and sea bass [8,10,11]. Yao et al. (2022) reported that the ultrasound-assisted salting of tuna fillets for 1 h (40 kHz, 840 W) and static salting for 2 h in 5% NaCl solution, respectively, resulted in the same salt content of the samples [3]. Moreover, Jin et al. (2023) reported that ultrasound-assisted salting (20 kHz, 350 W, 1 h) significantly increased the myogenic fiber fracture index and protein hydrolysis index of pork, which improved muscle tenderness [10]. Kang et al. (2017) demonstrated that 20 kHz ultrasound-assisted salting treatment significantly increased the proportion of water in the protein matrix within myogenic fibers and promoted oxidized myosin polymerization, which improved the WHC of beef [11]. Therefore, ultrasound-assisted salting pretreatment may be one of the methods to improve the quality of frozen tuna meat. However, ultrasound-assisted salting pretreatment has not been applied to frozen tuna meat processing.

Protein changes during muscle food processing and storage are closely related to quality characteristics, whereas conventional methods have limitations, such as gel electrophoresis only reflecting protein band intensity. Label-free quantitative proteomics, a high-throughput method to analyze and characterize the expression levels and sequence information of protein molecules and their derivatives, is now widely used to identify markers of quality changes and to reveal the mechanism of improvement of meat quality by processing methods [12,13,14]. Yu et al. (2023) used label-free proteomics to reveal a possible mechanism by which green tea extract coating improves the texture of refrigerated grass carp fillets by the modulation of the endogenous enzyme-induced degradation of myofibrillar proteins and the level of protein phosphorylation [13]. Zhang et al. (2022) found that sodium tripolyphosphate immersion improved the quality of frozen shrimp meat by maintaining the stability of cytoskeletal proteins and delaying muscle protein degradation with the method of label-free proteomics [14]. Even though an increasing number of improvement mechanisms of processing methods have been elucidated using proteomics, there is still a lack of information exploring the proteins’ molecular changes in UAS meat during frozen storage, which would help to optimize the processing methods and reveal the proteins’ molecular mechanisms of freshness preservation in frozen salted meats.

In this study, we investigated the effect of UAS and SS on the quality of frozen tuna fillets with a preliminary determination of physicochemical indices including water content, salinity, yield, color, texture, TVBN, TBARS, and WHC, as well as LF-NMR and histological methods. In addition, 4D label-free proteomic analysis with higher identification coverage and quantitative accuracy was utilized with the aim of fully elucidating the proteomic profile of UAS frozen fish fillets. This study will fill the gap in the content of UAS-processing strategies to improve the quality of frozen tuna meat, and provide a theoretical basis for further exploring the potential mechanisms of quality changes in cured meat products during frozen storage and for the better production of low-salt flavored fish products to satisfy consumer needs.

## 2. Materials and Methods

### 2.1. Sample Preparation

Yellowfin tuna (100–110 cm, 18–25 kg) were harvested from the South China Sea waters using pole-and-line fishing. After purchasing from a fishing company, the tuna were split into chunks, snap-frozen in liquid nitrogen and placed on dry ice for preservation, transported to the laboratory within 24 h, and preserved at −80 °C until use. After the removal of all visible adipose and connective tissues from the fish blocks, these fish blocks were cut into fillets of approximately 11 ± 1 g (3 × 3 × 1 cm^3^) at 4 °C, totaling 198 fillets. All fillets were randomized into three groups and processed immediately before completing the processing for the next step of frozen storage. All fillets were thawed at 4 °C for 2 h before testing unless otherwise noted. Moreover, all the chemicals and reagents used in the experiment were analytically reagents other than formic acid and acetonitrile which were chromatography reagents, and all chemicals and reagents were purchased from Sigma Chemical Co., Ltd. (Shanghai, China).

### 2.2. Salting and Ultrasound Pretreatment

The salting conditions were based on a previous study with minor modifications [2]. A 5% concentration of sodium chloride solution was used in the current study, and the ratio of brine and fish samples was set at 1:3 (*v*/*w*), which were placed together in a 100 mL beaker so that the fish fillets were leaning against the wall of the cup to be in full contact with brine. The temperature of the brine was at 4 °C, and the liquid level was higher than the fish fillets. An ultrasound cleaner (SB25-12DTD, Ningbo Xinzhi Bio-technology Co., Ltd., Ningbo, China) in 20 kHz single-frequency mode of operation was used, with the operating power set to 540 W. Ice water was added to maintain the operating temperature at 4 °C. In this study, untreated fish fillets that were left to stand at 4 °C for 30 min were used as the control group (NT), and the remaining groups were treated under the following conditions: standing salting at 4 °C for 30 min (SS) and ultrasound-assisted salting at 4 °C for 30 min (UAS). Immediately after 30 min of treatment, the samples of the three groups were removed from the saline solution, and the excess water on the surface was blotted with aseptic kitchen paper. The samples were individually packed in polyethylene bags after weighing and all were placed at −18 °C for storage for one week. The samples were thawed at 4 °C and then used for the determination of the indices.

### 2.3. Measurement of Physicochemical Indicators

#### 2.3.1. Yield, Water Content, and Salt Content

The determination of moisture content was based on the AOAC method [15]. One gram of the crushed sample was homogenized with 9 mL of distilled water in a disperser (T 25 digital ULTRA-TURRAX, IKA-Werke GmbH & Co., KG., Staufen, Germany), and the upper filtrate was collected. The salt content of the filtrate was measured with a hand-held salinometer (PAL-SALT, Atago Co., Ltd., Tokyo, Japan). The weights of the samples before (W_0_) and after (W_1_) salting were measured, and the yields were calculated with Equation (1):

Yield (%) = 100 × W_1_/W_0_ (1)

#### 2.3.2. WHC

##### Centrifuging Loss (CL)

The centrifuging loss was determined as described previously [4]. The samples were sliced into equal portions of about 1 g (2 × 1 × 0.5 cm^3^), put into a centrifuge tube with a double layer of slow-dosing filter paper, and centrifuged at 5000 rpm for 10 min at 4 °C. The centrifuging loss was calculated with Equation (2):

Centrifuging loss (%) = 100 × (W_3_ − W_4_)/W_3_ (2)

where W_3_ and W_4_ are the weights of the samples before and after centrifugation, respectively.

##### Thawing Loss (TL)

The thawing loss was provided as an index of the WHC of the samples and determined as described previously [2] and calculated with Equation (3):

Thawing loss (%) = 100 × (W_5_ − W_4_)/W_4_ (3)

where W_4_ and W_5_ are the weights of the samples before freezing and after thawing, respectively.

#### 2.3.3. Texture Properties and Color

The texture properties were determined following the method as described previously [16]. The fillets were cut into 1 × 1 × 1 cm^3^ pieces and then tested using a texture meter (TA. XT plus texture analyzer, Stable Micro Systems Co., Ltd., Surrey, UK). A P/50 probe was used to compress samples at a steady rate of 1 mm/s until 50% of the sample height was reached, held for 5 s, then repeated a second time. Experiments were repeated six times.

Thawed samples were photographed to show their visual appearance. The color characteristics of the samples were evaluated in terms of L* (lightness), a* (redness), and b* (yellowness) values using a chroma meter (CR-400, Konica Minolta Sensing Co., Inc., Tokyo, Japan).

#### 2.3.4. TBARS

The TBARS was determined as previously described with slight modifications [16]. Chopped sample (5 g) was weighed, added to 50 mL of 75 g/L trichloroacetic acid (TCA) mixture (1 g/L EDTA-2Na), homogenized at 10,000 rpm for 2 min, and then filtered. A 5 mL volume of the filtrate was mixed with the same volume of 0.02 mol/L thiobarbituric acid solution and heated in a 90 °C water bath for 30 min, and then, its absorbance was measured at 532 nm in 1 cm optical diameter cuvette after it cooled down. The TBA at a concentration of 0.1–1.0 μg/mL was used to create a standard curve, and the TBARS was expressed as the malondialdehyde content (mg MDA/kg) of the sample.

#### 2.3.5. TVBN

The TVBN was determined as described previously [17]. Briefly, 10 g of chopped sample was mixed with 100 mL distilled water and homogenized, stirred for 30 min, then filtered. A 1 g quantity of MgO was added to 10 mL of the filtrate and analyzed using an automatic Kjeldahl nitrogen analyzer (Kjeltec 2100, Foss Co., Corp., Hilleroed, Denmark) after mixing adequately. The TVBN was expressed as milligrams of N per 100 g of fish.

### 2.4. LF-NMR and Magnetic Resonance Imaging (MRI)

Moisture distribution and proportions of the samples were evaluated using a LF-NMR analyzer with a 20 MHz frequency (NMI20-040H-I, Shanghai Niumag Analytical Instruments Co., Inc., Shanghai, China). The samples were cut into 1.5 × 2 × 2 cm^3^ blocks, wrapped in polyethylene films, and then thawed. The transverse relaxation time (T_2_) of samples was measured using CPMG sequence, and the pulse parameters were as follows: SW = 200 kHz, TW = 3000 ms, NS = 4, TE = 0.12 μs. Each measurement was repeated 3 times. The pseudo-color images of the proton density of samples were obtained with MRI software (Niumag NMR Imaging System V3.0) analysis.

### 2.5. Microstructural Observation

#### 2.5.1. Light Microscopy (LM)

Thawed samples were fixed at 4 °C for 24 h and then embedded, sectioned, stained with hematoxylin–eosin (H&E), and finally observed under a light microscope (Eclipse ci, Nikon Instruments Co., Inc., Tokyo, Japan). For frozen samples, the isothermal freeze replacement technique was used for preparation.

#### 2.5.2. Scanning Electron Microscopy (SEM)

Samples were cut into 0.5 × 0.3 × 0.3 cm^3^ sections and fixed for 4 h in 2.5% glutaraldehyde and 0.1 mol/L phosphate buffer (pH 7.4). After being dehydrated in a series of ethanol solutions, the samples were dried, coated with gold, and then observed with a scanning electron microscope (Regulus 8100, HITACHI Scientific Instruments Co., Ltd., Tokyo, Japan).

### 2.6. Proteomic Analysis

#### 2.6.1. Protein Extraction and Digestion

Frozen fish samples were ground to a powder under liquid nitrogen and then mixed with 4 times the volume of lysis buffer (8 mol/L urea, 1% sodium dodecyl sulfate, and protease inhibitor). After 30 min on ice (5–10 s of shaking every 5 min), the mixture was centrifuged at 16,000× *g* for 30 min at 4 °C, and the supernatant protein concentration was determined using the BCA method.

A 100 μg quantity of protein was added to triethylammonium bicarbonate buffer (100 mmol/L final concentration) and mixed with tris (2-carboxyethyl) phosphine (10 mmol/L final concentration) to enable a reaction for 60 min at 37 °C, and then mixed with iodoacetamide (40 mmol/L final concentration) for reaction at room temperature for 40 min in the dark. Then, the mixture was mixed with six times the volume of the pre-cooled acetone and incubated at −20 °C for 4 h, followed by centrifugation at 10,000× *g* for 20 min at 4 °C. The precipitate was collected and dissolved in 100 mL of acetone. The precipitate was collected and dissolved in 100 μL of 100 mM triethylammonium bicarbonate buffer, then digested overnight at 37 °C with trypsin at a substrate-to-trypsin ratio of 50:1 (*w*/*w*). The digested peptides were dried under vacuum, re-dissolved in 0.1% trichloroacetic acid, desalted using 30 μm Oasis HLB 96-well plate (Waters, Massachusetts, USA), and then quantified using the PierceTM peptide quantification assay kit (Cat. 23275, Thermo Fisher, Shanghai, China).

#### 2.6.2. LC-MS/MS Analysis

Digested peptides (0.1 μg/μL) were analyzed using an Easy-nLC 1000 system (Thermo Fisher Scientific, Waltham, MA, USA) coupled with a timsTOF Pro2 mass spectrometer (Bruker, Karlsruhe, Germany). The peptides were loaded onto a C18 column (75 μm × 25 cm × 1.6 μm, Thermo Fisher, Waltham, MA, USA) at 300 nL/min, and eluted with solvent A (0.1% formic acid and 2% acetonitrile) and solvent B (0.1% formic acid and 80% acetonitrile) over 60 min (0–45 min: 3% solvent B; 45–50 min: 28% solvent B; 50–55 min: 44% solvent B; 55–60 min: 90% solvent B). The separated samples were identified using a timsTOF Pro2 mass spectrometer in DDA mode with the MS scanning range set at 100–1700 *m*/*z*.

#### 2.6.3. Protein Identification and Bioinformatics Analysis

The original data produced from LC-MS/MS were imported into MaxQuant software (version 2.0.3.1). The software search parameters were as follows: Cys alkylation, iodoacetamide; enzyme name, trypsin/P; the maximum number of missed cleavages, 2; precursor mass tolerance, 10 ppm; dynamic modification, oxidation (M) and acetyl (protein N-terminus); and static modification, carbamidomethyl (C). The data was filtered with matched unique peptide ≥1 and a false discovery rate (FDR) of below 0.01.

Two comparisons were made, i.e., SS vs. NT and UAS vs. NT. Proteins with a fold change (FC) >1.3 or <1/1.3 and *p* < 0.05 were considered significant DAPs. Functional annotations for DAPs were analyzed using three databases: Gene Ontology (GO), Kyoto Encyclopedia of Genes and Genomes (KEGG), and NCBI non-redundant protein library (NCBI-NR). The highest-ranked hit to a homologous protein in the NCBI database was selected for identification, and then, a BLAST sequence similarity search was run for the protein sequences in the UniProt database to evaluate the reliability of the identification results.

### 2.7. Statistical Analysis

Data were examined for homogeneity and normal distribution by using Levene’s test and Shapiro–Wilk test, respectively. All measurements were carried out in triplicate (*n* = 3), and the data were presented as the mean ± standard deviation (SD). Data were evaluated using one-way ANOVA analysis with Duncan’s multiple comparisons test. Significant differences at *p* < 0.05 calculated from the determinations were analyzed using the SPSS software 26.0 (SPSS Inc., Chicago, IL, USA).

## 3. Results and Discussion

### 3.1. Changes in Physicochemical Indicators of SS and UAS

#### 3.1.1. Changes in Quality-Related Indicators of SS and UAS

The water content, salt content, and yield were significant indicators for assessing salting effects. Among the three groups, the water content, salt content, and yield of the SS group increased significantly (*p* < 0.05) compared to those of the NT group (Table 1), while these indicators of the UAS group increased significantly compared to those of the SS group, which indicated that UAS enhanced the effect of salting in fillets. In general, osmotic pressure is involved in regulating the dynamic balance of water and salt concentration inside and outside cells, and limiting the permeability of the salt in cell membrane [11]. However, the formation of microjets and mechanical squeezing using ultrasonic cavitation enhanced the permeability of cell membranes and facilitated the transport of substances between brine and muscle, which may be the main reason for the better salting effect of UAS. Notably, Yao et al. (2022) reported that the high efficiency of ultrasound in mass transfer is mainly achieved by the cavitation and sponge effects [3], which partially explains this phenomenon. Moreover, Bai et al. (2023) also reported that the UAS group (500 W, 30 min) was 1.3 times the salt content of the SS group, and UAS significantly enhanced the curing efficiency of sea bass [8].

**Figure 1:**
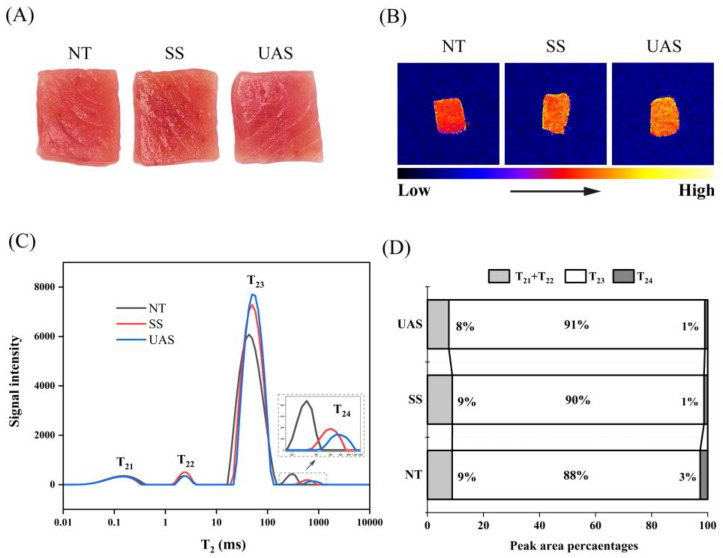
Changes in visual appearance (**A**) and moisture distribution of tuna fillets treated with SS and UAS. Images of (**B**) T_2_-weighted magnetic resonance; (**C**) lateral relaxation time (T_2_); and (**D**) relative water content.

Color is an essential indicator of tuna quality that influences consumers’ intention to purchase tuna [16]. Compared with the NT group, the edges of the SS tuna fillets lightened in color, while the surface showed a glossy appearance (Figure 1A). Compared with NT and SS groups, the a* and b* values of the UAS group were significantly decreased (*p* < 0.05), indicating that the color of UAS fillets was more susceptible to freezing pressure (Table 1). As expected, changes in a* values from the color indices were mainly attributed to the oxidation of myoglobin, and the b* value is associated with yellow pigment related to lipid oxidation [18]. These results suggest that UAS has a greater effect on the special red color of frozen fish fillets. According to Jiang et al. (2022), the lipid and protein in the salted tuna fillets might be more susceptible to oxidation during frozen storage when the endogenous antioxidant systems in muscle become unbalanced [19]. Pan et al. (2022) reported that ultrasonic cavitation accelerated the excessive accumulation of free radicals, leading to the oxidative browning of myoglobin in UAS pork samples [20].

Textural property is the key attribute for evaluating tuna tenderness, including hardness, springiness, and adhesiveness. In the current study, the results of the SS group and the UAS group showed a trend of significant increase in springiness and a trend of significant decrease in the hardness of groups (*p* < 0.05) (Table 1) compared to those of the NT group, indicating that the salted tuna fillets became tender. Interestingly, it has been explained by the results of Du et al. (2021) that showed changes in tenderness in muscle are mainly caused by structural changes in myofibrillar and connective tissues [21]. In addition, the adhesiveness was significantly increased in the UAS group compared to that of the NT group (*p* < 0.05), suggesting that UAS led to moderate protein oxidation. According to a previous study [7], it was a possible reason for the increased adhesiveness in the UAS group that resulted in the ultrasound-induced formation of a sticky protein gel on the meat surface from salt-soluble myofibrillar proteins.

The WHC is an important index to evaluate the ability of muscles to retain water. Compared with the NT group, the centrifuging loss and thawing loss of the UAS group were significantly decreased (Table 1), and the centrifuging loss of the UAS group was lower than that of the SS group (*p* < 0.05), indicating that UAS significantly improves the WHC of frozen fish. Mass transfer efficiency and protein changes may be the potential reasons for the increased WHC in UAS group. Generally, ultrasound promoted the mass transfer efficacy of saline in muscle fibers, achieving broader hydration and higher water–protein interactions, with more water and NaCl molecules attached near myofibrillar proteins [11]. On the other hand, Jin et al. (2023) reported that the coagulation, denaturation, oxidation, and cross-linking of proteins in pork muscle leads to the formation of a special gel network under the combined action of ultrasound and brine [10], which might be the other factors that cause the improvement in the WHC. This evidence id partly supported by our hypothesis.

The freshness of fish is assessed using TVBN, which is a critical indicator [16]. The TVBN values were significantly decreased (*p* < 0.05) (Table 1) in the SS group compared with those in the NT group, and in the UAS group compared with those in the SS group (*p* < 0.05). The results suggested that UAS can better reduce the production of amines during fish storage and contribute to the extension of shelf life. In general, the increase in TVBN value may be due to the accumulation of the volatile, small-molecule amine compounds from the degradation of nitrogen-containing compounds and proteins induced by enzymes and bacteria [17]. The changing trend of TVBN was essentially the same as reported by Yao (2022), and it was found that tuna fillets treated with ultrasound-assisted salting (840 W, 5% NaCl, 1 h) had a significantly lower TVBN value than that of no-salted fillets after one freeze–thaw cycle [22].

As a byproduct of lipid peroxidation in meat, malondialdehyde (MDA) is a main indicator to evaluate the degree of lipid oxidation, which is represented by TBARS values [16]. Our results showed that the TBARS values were significantly increased (*p* < 0.05) (Table 1) in the UAS group compared with those in the NT group (*p* < 0.05), suggesting that UAS promoted the lipid oxidation process in salted fish fillets. It was confirmed that low NaCl content (<2.5%) could promote lipid oxidation because NaCl causes hemoglobin and myoglobin to release the pro-oxidant ferric ions and inhibit antioxidant enzyme activity [7,18]. The increase in TBARS levels in the UAS group may be due to ultrasound, affecting antioxidant enzyme activity or cell membrane permeability and facilitating oxidant–substrate contact, which has not been conclusively investigated yet.

#### 3.1.2. Changes in LF-NMR and MRI of SS and UAS

LF-NMR was used to evaluate the distribution and migration of water in fish meat by measuring proton transverse relaxation time (T_2_) [11]. There were three distinct water populations identified as characteristic peaks: T_21_ + T_22_ (<10 ms), T_23_ (10–100 ms), and T_24_ (>100 ms) (Figure 1C). Results showed that there were no significant differences in all groups on the peak area of T_21_ + T_22_. Generally, T_21_ + T_22_ is identified as water combined with protein side chains and macromolecular [11], so there is high stability on T_21_ + T_22_. Furthermore, the area and proportion of the T_24_ peak in the UAS and SS groups were significantly smaller than those in the NT group (Figure 1C,D), suggesting that SS and UAS impeded water migration from T_23_ to T_24_ and that more free-flowing water was retained on the outside of myofibrils. Commonly, T_23_ is recognized as water in the extracellular space and water in the intra- and extra-myofibrillar protein matrix [4]. The changes in T_23_ showed that UAS and SS resulted in more water retention around the myogenic fibers, leading to better elasticity and juiciness properties of the fish fillets, which was consistent with a previous study [11].

MRI can intuitively reflect water distribution in meat after frozen storage. As shown in Figure 1B, the yellow area indicates a higher signal intensity of hydrogen protons and more water, while the blue area indicates less water [10]. The higher signal intensity of hydrogen protons in the SS and UAS groups compared to that in the NT group indicated that SS and UAS groups retained water better, which may be related to improved hydration due to oxidative modification. Pan et al. (2022) reported that 6% NaCl saline treatment induces oxidative modifications in pork proteins to improve hydration [20]. In addition, the yellow areas in the UAS group were more uniformly distributed compared to the SS group, suggesting that the migration and distribution of water in the fish were significantly improved under the influence of ultrasound. We speculate that this may be related to cavitation effects. It was extensively reported that ultrasound promotes the diffusion of NaCl in muscle tissue, mainly through cavitation effects [10,11]. Yao et al. (2022) found that microjets formed by ultrasonic cavitation induce an increase in the effective diffusion rate of water and NaCl in tuna muscle, which supports our hypothesis [3]. Notably, Wu et al. (2021) found the T_23_ area significantly decreased from 0.5 h to 1 h in the brine injection group, and saline injection requires more time to adequately diffuse salt in the tissue of grass carp [9]. In comparison, our MRI results indicated that UAS may be a potentially better method than saline injection in improving salting efficiency, which needs to be demonstrated in future studies.

### 3.2. Changes in Histological Observations of SS and UAS

**Figure 2:**
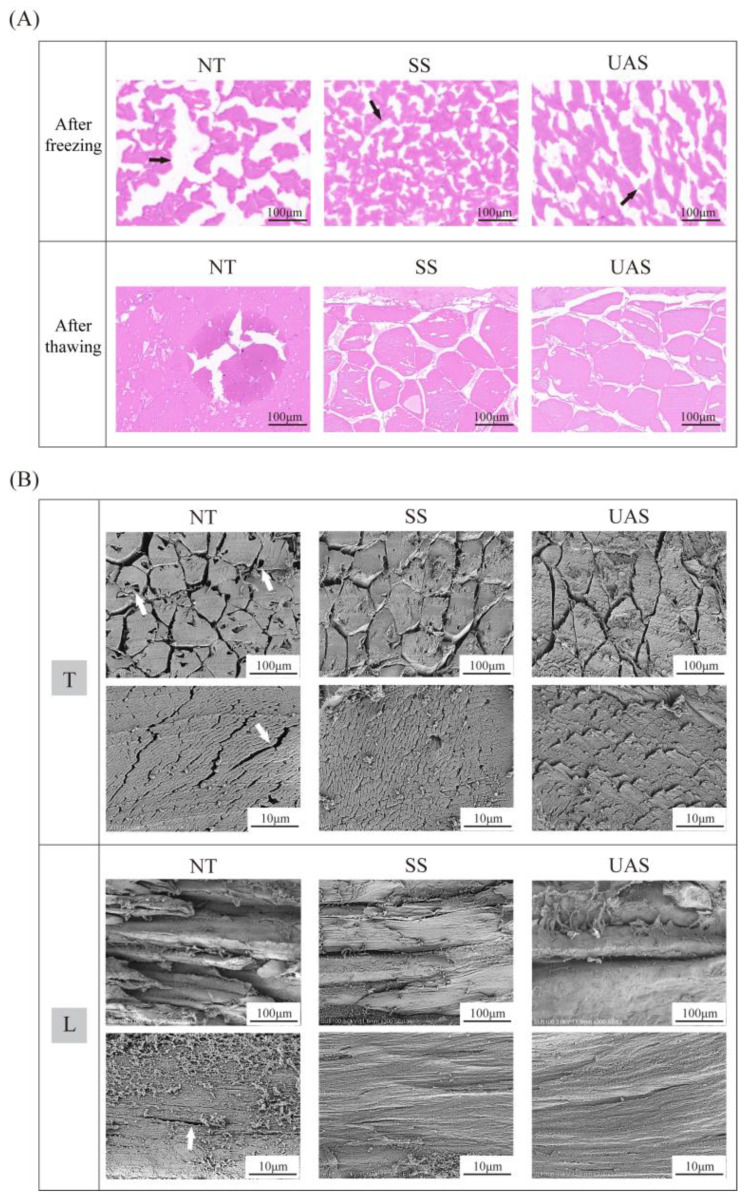
The microstructure of tuna fillets from NT, SS and UAS groups was observed using LM (**A**) and SEM (**B**). (**A**) After freezing: the H&E staining of cryosections; after thawing: the H&E staining of thawed sections. (**B**) T: transverse sections; L: longitudinal sections. Black arrows indicate ice crystals of different shapes, and white arrows indicate fissures or pores.

The changes in frozen fish tissues (H&E staining) are shown in Figure 2A with LM. The white voids observed in frozen tissues could reflect the morphology of ice crystals [2]. It was clear that the shapes of ice crystals in the NT group were more irregular and larger when compared with those in the SS and UAS groups after freezing. Normally, the ice crystals tend to become larger due to the cryoconcentration during freezing, resulting in severe extrusion to the surrounding cells [4], which was the reason that caused the prominent regions of frostbite observed in the NT group after thawing. In general, smaller ice crystals are a prerequisite for the cells to return to normal morphology after thawing [2]. We observed that the tissues in the SS and UAS groups had a full morphology with clear borders and smaller ice crystals compared with that in the NT group after thawing. Tan et al. (2021) reported that large ice crystals disrupted tissue integrity and severely damaged protein conformation [4], which is consistent with our results. Moreover, it is worth noting that the formation of ice crystals was more continuous in the UAS group after freezing, and the extracellular space was enlarged in the UAS group, which may be related to degraded connective tissue in the extracellular matrix. Du et al. (2021) found that the cavitation effect of ultrasound (500 W, 30 min) on the muscles of chicken weakens their connective tissue structure, promotes their proteolysis and disrupts the molecular cross-linking of collagen [21].

The microstructure of muscle fibers in thawed salted fish tissue is shown in Figure 2B by SEM. The SEM result showed that myofibers in the NT group contracted after water loss, and their structure was impaired, with larger pores and fissures on the surface of myofibers and significant extracellular voids. Conversely, the myofibers of the SS and UAS groups arranged tightly and orderly, and their surface hollows almost disappeared, with the extracellular voids occupied by enlarged myofibers. The morphological performance of myofibers of the SS and UAS groups contributed to improving the recoverability of thawed muscle, indicating less damage by ice crystals. We hypothesized that muscle damage from ice crystals may be related to the altered protein structure. Tan et al. (2021). reported that mechanical damage caused by ice crystals accelerates protein degradation and affects water reabsorption in thawed tissues [4], which supported our hypothesis. Moreover, we also noticed that the myofiber diameters of the SS and UAS groups showed morphological changes compared to those of the NT group. According to the available research, the morphological enlargement of myofibrils in low-salt salting meats was reported to be influenced by several factors [2,6,21]: (1) the myofibrillar proteins are solubilized/extracted in the salt solution, and protein–water interactions enhanced; (2) Cl^−^ ions bind to myofilaments, which enhances electrostatic repulsion and the swelling of the filament lattice; and (3) salting softens/damages the pericellular connective tissue, including the perimysium and the endomysium. Notably, the myofibers in the UAS group exhibited an excessive increased in size, the perimysium was broken, and the cell edges showed obvious fragmentation and breakage, which indicated that ultrasonic cavitation subjected the surface of the myofibers to violent shocks and oscillations. Jin et al. (2023) reported that ultrasonic cavitation generated by ultrasound-assisted salting (20 kHz, 350 W for 1 h) dissociated the myofibers of pig *biceps femoris* with the high hydrolysis/rupture of proteins, causing the more pronounced swelling of muscle microstructures [10]. Overall, it is a reasonable speculation that acoustic cavitation disrupts collagen cross-links in the cell matrix and the endomysium, and salt-solubilized myofibers are free to expand, while the filament lattice is further swollen.

### 3.3. Changes in Proteomics Analyses of SS and UAS

#### 3.3.1. Identification of Proteins

**Figure 3:**
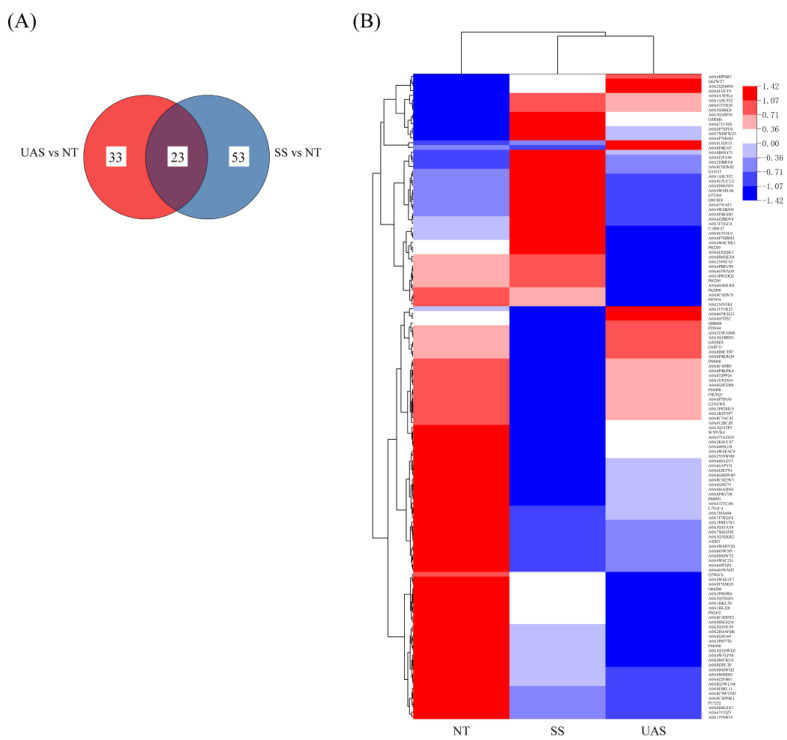
(**A**) Venn diagram of the DAPs identified using 4D label-free proteomics in the SS vs. NT and UAS vs. NT comparisons. (**B**) Hierarchical cluster heat map of DAPs. The rows represent individual proteins with UniProt numbers located to the right of each corresponding row. Different colors represent different relative abundance levels of proteins, where blue represents higher intensity, and red represents lower intensity.

4D label-free quantitative proteomics analysis was performed to evaluate the protein expression level and further clarify the potential association between key proteins and the quality properties of frozen tuna fillets treated with SS and UAS pretreatments. A total of 781 proteins were identified, of which 658 identified proteins (84.25%) had molecular weights (MW) in the range of 1–60 kDa, in addition to a peptide length predominantly ranging from 7 to 22 amino acids. Moreover, a total of 129 of the identified proteins (92.81%) had sequence coverage of ≥60%, which matched those reported in the NCBI-NR database and the UniProt database. The results of the above data suggested the validity and reliability of the operational procedures performed. According to FC >1.3 or FC <1/1.3 (*p* < 0.05), there were 24 up-regulated proteins and 52 down-regulated proteins in the SS vs. NT comparison (Table S1), and 10 up-regulated proteins and 46 down-regulated proteins in the UAS vs. NT comparison (Table S2). As illustrated by the Venn diagram (Figure 3A), 23 shared DAPs were identified in both groups, along with 53 non-shared DAPs from the SS vs. NT comparison and 33 non-shared DAPs from the UAS vs. NT comparison. Furthermore, the hierarchical cluster heat map (Figure 3B) showed a considerably distinct proteomic profile in the three groups, indicating significant changes in the proteomic trends of fish proteins in the SS and UAS groups after frozen storage, and the meaningful data analysis for DAPs can be performed.

#### 3.3.2. GO Annotation Analysis

**Figure 4:**
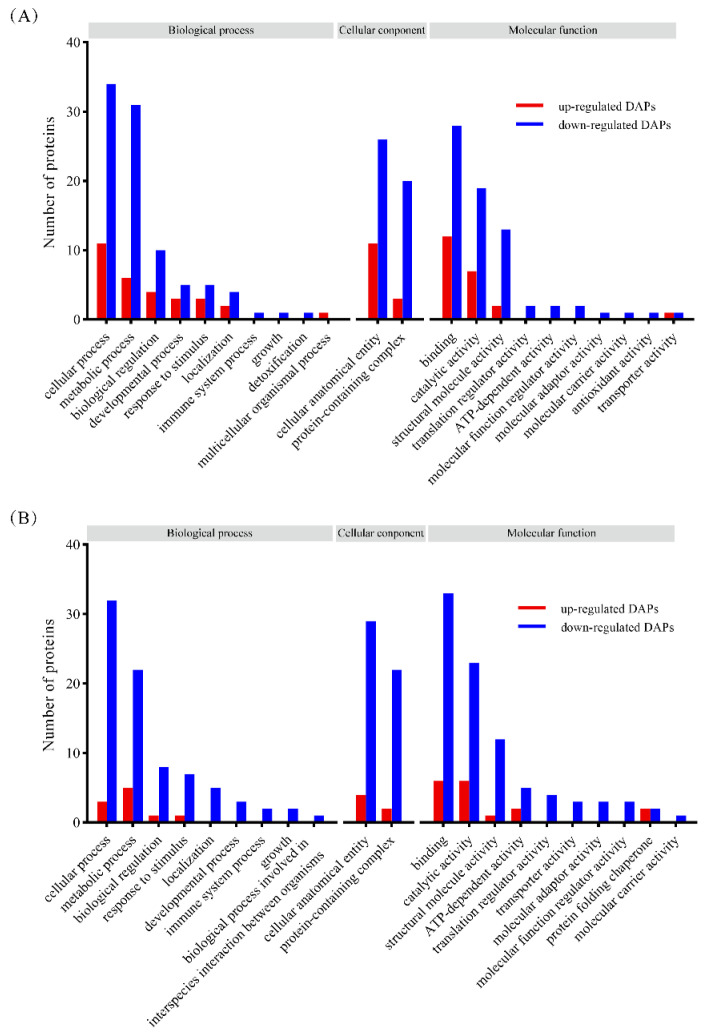
GO annotation analysis of the DAPs identified using 4D label-free proteomics in the (**A**) SS vs. NT and (**B**) UAS vs. NT comparisons. Red and blue colors represent the higher and lower abundance levels of the identified DAPs in the tuna muscle, respectively.

To explore the biological functions of DAPs potentially associated with meat quality, the GO analysis of the identified DAPs in two comparisons (SS vs. NT and UAS vs. NT) was performed to determine their enriched biological process, molecular function, and cellular component. For the biological process, DAPs in the SS vs. NT and UAS vs. NT comparisons mainly focused on cellular process (GO: 0009987), metabolic process (GO: 0008152), and biological regulation (GO: 0065007) (Figure 4). For the cellular component, DAPs in the SS vs. NT and UAS vs. NT comparisons focused on the cellular anatomical entity (GO: 0110165) and the protein-containing complex (GO: 0032991) pathway. Moreover, for the molecular function, DAPs in the SS vs. NT and UAS vs. NT comparisons focused on binding (GO: 0005488), catalytic activity (GO: 0003824), and structural molecular activity (GO: 0005198). Overall, most of the DAPs in the two comparisons were focused on the same GO-annotated pathways, with a few embodied in different pathways, suggesting that the two processing methods are similar in affecting the molecular biological functions of muscle proteins. Notably, DAPs associated with these biological annotations play an important role in maintaining the structure and function of muscle tissues during frozen storage. According to Zhang et al. (2020), the GO annotation of freeze-stored peeled shrimp (*Litopenaeus vannamei*) results showed that DAPs related to muscle stability were enriched in the cellular process, protein-containing complex, and binding [14], which is in agreement with our results. We selected some interesting proteins for functional analysis that will be performed in subsequent panels.

#### 3.3.3. KEGG Pathway Analysis

**Figure 5:**
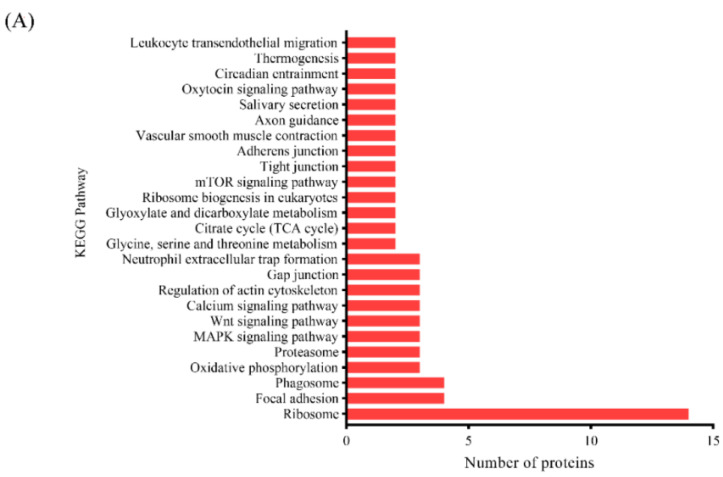

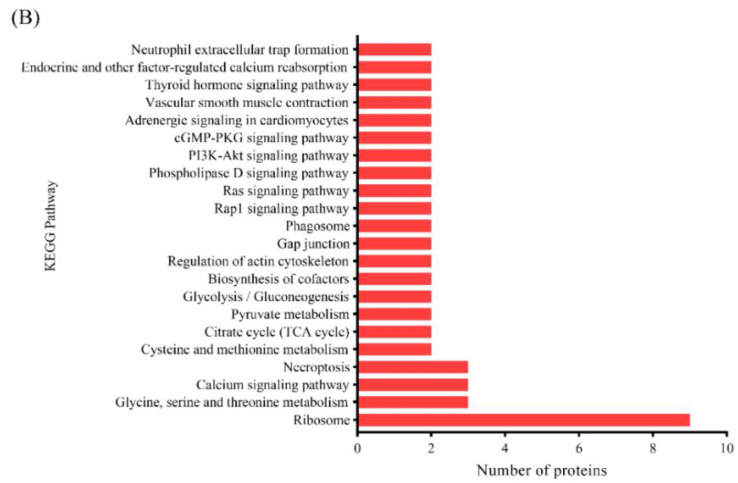
KEGG pathway analysis of the DAPs identified using 4D label-free proteomics in the (**A**) SS vs. NT and (**B**) UAS vs. NT comparisons.

KEGG pathway analysis was performed to analyze the biological pathway of the identified DAPs in two comparisons (SS vs. NT and UAS vs. NT). There were 25 biological pathways related to DAPs of the SS vs. NT comparison (Figure 5), which included ribosome, focal adhesion, phagosome, oxidative phosphorylation, proteasome, and the regulation of actin cytoskeleton, with ribosome being the most enriched of these pathways (*p* < 0.05). The ribosome pathway diagram is shown in Figure S1. In addition, there were 22 biological pathways related to DAPs of the UAS vs. NT comparison, which included ribosome, amino acid metabolism (glycine, serine, and threonine metabolism), calcium signaling pathway, necroptosis, and glycolysis, with glycine, serine, and threonine metabolism being the most enriched of these pathways (*p* < 0.05). The glycine, serine, and threonine metabolism pathway map is shown in Figure S2. Ribosomal proteins are responsible for ribosome assembly and protein translation. It was reported that ribosomal proteins are prone to degradation/oxidation when experiencing metabolic or external stresses such as cold, mechanical damage, and free radical attack [23,24]. Multiple ribosomal protein subunits were accumulated at lower abundance levels in the SS vs. NT and UAS vs. NT comparisons, which may be caused by freeze-induced oxidation and/or degradation induced by reactive oxygen species (ROS). The above changes in ribosome abundance agreed partially with the previous findings of frozen peeled shrimp (*Litopenaeus vannamei*) [14]. Moreover, it was reported that focal adhesion is associated with the site of structural attachment between the extracellular matrix and the cell membrane, and the degradation of adhesion-associated proteins affects cytoskeletal structure, intracellular signaling pathways, and drip channel formation [12]. In the SS vs. NT comparison, most DAPs associated with focal adhesion showed a trend of increased abundance, indicating that salting may contribute to better cytoskeletal structure and less drip channel by minimizing mechanical damage from ice crystals. Hou et al. (2020) found more adhesion-related proteins were disrupted and released in pork with high drip loss [12]. Particularly, the necroptosis pathway appeared in the UAS vs. NT comparison, which is considered to be a passive degenerative phenomenon induced by physical injury or other unfavorable environmental factors, and as apoptosis is regulated by adenosine triphosphate (ATP) levels [25], the result indicates that UAS pretreatment might promote the process of cell necroptosis in tuna fillets during frozen storage.

#### 3.3.4. Analysis of DAPs Associated with Quality Changes

##### Changes in DAPs Associated with Protein Structure

In this study, most DAPs were identified to be associated with biological functions such as protein structure, protein turnover, and energy metabolism. In SS vs. NT comparison, the abundance of filamin-B, filamin-C, fibrillin-1, and titin-like isoform X15 showed up-regulation, and the abundance of myosin heavy chain 10 and troponin I showed down-regulation (Table S1). The decreased expression of myosin heavy chain 10 and troponin I indicated degradation driven by salting and freezing, which is consistent with the previous findings [26]. Moreover, as an important component of elastic fibers in connective tissue, the reduced levels of fibrillin-1 indicated the disruption of collagen cross-links in the extracellular matrix after salting, which is in agreement with our SEM results and a previous study [2]. 

On the other hand, the abundance of myosin heavy chain 10, troponin I, myosin tail domain-containing protein, and myosin-7-like showed down-regulation in the UAS vs. NT comparison. These myosin-related proteins showed reduced abundance, suggesting that myosin filaments in the muscle of the UAS group may be denatured/degraded during frozen storage. Shui et al. (2022) reported that the abundant of DAPs associated with structure including troponin I and myosin heavy chain decreased in the muscle of frozen hairtail (*Trichiurus lepturus*), which may be related to freeze-induced degradation, enzymatic actions, and oxidation caused by reactive radicals [24]. The decreased abundance of troponin in UAS vs. NT comparison was also explained by the result of a previous study [24]. Noteworthily, the fact that the degradation and/or degradation of myosin filaments may be associated with the increased WHC. It has been reported that myosin filaments and actin filaments slide past one another and partially overlap when sarcomeres contract, altering the myofilament lattice spacing, and causing the sarcoplasm to be expelled to the surface of meat in the form of drip [27]. When motor myosin filaments are degraded, the diastolic muscle segments facilitate water retention due to electrostatic repulsion, capillary forces, and protein–water interactions, which support our WHC results. 

##### Changes in DAPs Associated with Energy Metabolism

The abundance of ATP-synt_ab_N domain-containing protein and cytochrome c oxidase (CCO) subunit showed up-regulation in the SS vs. NT comparison. As the terminal enzyme of the electron transport chain (ETC) in the inner membrane of mitochondria, CCO is involved in ATP production, which generates ROS [28]. These two DAPs are involved in oxidative phosphorylation. Interestingly, a significant decrease in catalase abundance was found in the SS vs. NT comparison. Catalase quenches excess ROS to prevent cellular peroxidation [29]. The down-regulation of catalase means that protein in salted muscle suffers more oxidative damage, and NaCl promotes intracellular oxidation by affecting the activity of antioxidant enzymes, which is partially supported by our TBARS result, and consistent with previous findings [26]. It is reasonable to speculate that salted fish are primarily attacked by excess ROS produced mainly by oxidative phosphorylation. However, the oxidative reactions that occur during freezing are complex and deserve further study. 

In the UAS vs. NT comparison, the abundance of glucose-6-phosphate isomerase, ADP/ATP carrier protein (AAC), ATP synthase subunit beta, adenylosuccinate synthetase, fumarate hydratase, and the acetyltransferase component of pyruvate dehydrogenase complex showed down-regulation. The ATP synthase is involved in the process of coupling electron transport to the activity of complex V during ETC to generate ATP, and its decreased expression affects the H^+^ gradient, resulting in electron leakage. Changes in electron flux and ATPase activity mediate the conversion of oxygen monoatoms to hydrogen peroxide [30]. In general, molecules involved in glycolysis and pyruvate metabolism undergo the TCA cycle to generate substrates that enter the ETC to participate in oxidative phosphorylation. Acetyl coenzyme A serves as an entry point to the TCA cycle and is involved in the important aspects of the glycolysis and pyruvate metabolic pathways, and its altered abundance affects the reduction of NAD^+^ to NADH, whereas NADPH/NADP^+^ renews the antioxidant systems with its reductive potential [30]. We speculate that the oxidation/degradation of several enzymes involved in the ETC is one of the reasons for the dysregulation of ROS, and another possibility is that small molecule autoxidation generates excess ROS, which is a by-product of the cavitation effect of ultrasound [20,28]. Notably, the abundance of DAPs associated with the color change showed a decreasing trend in the UAS vs. NT comparison (Table S2), including coproporphyrinogen oxidase (CPO) and myoglobin (Mb). It was reported that the meat color state is determined by the concentration and redox state of myoglobin, hemoglobin, and other pigments [16]. The decreased abundance changes in CPO and Mb indicated the significant oxidative denaturation of the pigmented proteins in the UAS group during frozen storage, contributing to the fading of meat color, which is consistent with a lower a* value (Table 1).

##### Changes in DAPs Associated with Protein Turnover

Some DAPs involved in proteasome were significantly down-regulated in SS vs. NT comparison, including E2 ubiquitin-conjugating enzyme, peptidase S1 domain-containing protein, three 20S proteasome subunit (α1, α3, α5) (Figure S3). The 20S proteasome subunit alpha-type is a component of the 26S proteasome, which is involved in the ATP-dependent degradation of ubiquitinated proteins and its activity is regulated by phosphorylation modifications [31]. In the previous analysis of DAPs, we found that the oxidative phosphorylation pathway was dysregulated in SS vs. NT group, which may be responsible for 20S proteasome degradation. Moreover, E2 ubiquitin-conjugating enzymes are responsible for identifying lysine residues of ubiquitin chain substrates and determining ubiquitin chain extension [32]. The reduced abundance of E2 ubiquitin-conjugating enzymes may be due to oxidative modification of lysine residues in the UAS group during frozen storage, which is in agreement with the previous findings [26].

In the UAS vs. NT comparison, the abundance of calpain small subunit 1-like showed up-regulation, and the abundance of heat shock protein HSP 90-alpha (HSP90-α) and protein kinase C alpha type showed down-regulation. Calpain mainly functions based on the degradation of various muscle proteins, especially in myofibrils [33]. A decreased abundance of calpain small subunit 1-like indicated that the overexpression of calpain may occur after UAS treatment, followed by degradation in the high ionic strength environment during frozen storage. Li et al. (2023) reported that the myofilaments of silver carp (*Hypophthalmichthys molitrix*) were progressively disassembled/destroyed at high ionic strengths, while a decrease in the abundance of calpain was observed [34]. Moreover, it was reported that the perturbation of the protein degradation dysregulates cell cycle proteins, which tends to promote apoptosis [33]. Interestingly, as chaperone proteins involved in anti-apoptotic effects, heat shock proteins protect structural proteins such as tubulin and actin from denaturation [35]. Heat shock protein HSP90-α and microtubule-associated protein were down-regulated in the UAS vs. NT comparison, suggesting that the regulation of oxidative stress and apoptosis may be affected in the muscle of the UAS group. Hou et al. reported lower antioxidant stress and anti-apoptotic capacity in pork with high levels of glycolysis [12]. Moreover, HSP90 synergizes with the unc-45 myosin chaperone B (UNC45) protein to act as a myofilament assembly factor, and their lower abundance in the UAS group indicated that myofilament assembly behavior is affected, and its structural stability is reduced. Ge et al. (2018) found a negative correlation between the reduction of UNC45 and the dissociation of actin in ice-stored grass carp muscle (*p* < 0.05), and that the dissociation of the myofilaments may contribute to the increase in tenderness [5]. Based on our results, we found that UAS pretreatment more or less promoted necroptosis/apoptosis, weakened contractile regulation of myofibrils, and triggered a series of enzyme-activated cascade reactions.

## 4. Conclusions

In this study, a comprehensive analysis of UAS and SS tuna fillets was carried out from three aspects, i.e., physicochemical indices, histological observations, and proteomic analyses, in an attempt to provide a theoretical basis for the development of novel UAS frozen fish processing methods. Compared with untreated fillets, UAS significantly increased the yield, springiness, WHC, and T_23_ value of the fillets and reduced unfavorable proteolysis. Notably, UAS promoted the structural disintegration of myogenic fibers and the expansion of the extracellular space, allowing proteins to bind with more water. Additionally, 76 and 56 DAPs in the SS vs. NT and UAS vs. NT comparisons, respectively, were identified using 4D label-free proteomics, mainly related to tissue structure, energy metabolism, and protein turnover. Myoglobin, calpain small subunit, myosin heavy chain, and other key proteins closely associated with quality changes showed decreased expression in the UAS vs. NT comparison. Nevertheless, this study still has some limitations, mainly the difficulty in controlling the color degradation of UAS fillets after frozen storage, which is related to the free radical-induced oxidation. Thus, we will investigate the effect of suitable ultrasonic parameters to optimize the whole UAS method in subsequent studies.

## Figures and Tables

**Table 1:** Quality-related indicators of tuna fillets treated by SS and UAS pretreatments.

Quality Parameters	NT	SS	UAS
Water content (%)	71.48 ± 0.10 c	73.81 ± 0.09 b	74.44 ± 0.18 a
Salt content (%)	1.10 ± 0.02 c	2.01 ± 0.02 b	2.10 ± 0.02 a
Yield (%)	98.49 ± 0.44 c	104.76 ± 0.31 b	106.47 ± 0.26 a
L*	37.82 ± 1.00 a	36.53 ± 0.04 a	37.32 ± 0.92 a
a*	9.10 ± 0.32 a	8.06 ± 0.11 b	6.83 ± 0.14 c
b*	2.52 ± 0.24 a	1.21 ± 0.09 b	0.64 ± 0.04 c
Hardness (g)	570.99 ± 5.41 a	523.15 ± 7.37 b	535.80 ± 11.24 b
Springiness	0.51 ± 0.01 b	0.59 ± 0.02 a	0.59 ± 0.02 a
Adhesiveness (g·sec)	6.49 ± 0.86 b	7.37 ± 1.40 ab	11.24 ± 1.73 a
CL (%)	31.30 ± 1.36 a	27.71 ± 2.40 b	24.09 ± 1.14 c
TL (%)	1.36 ± 0.12 a	0.87 ± 0.09 ab	0.65 ± 0.11 b
TBARS (mg MDA/kg)	0.74 ± 0.07 b	0.89 ± 0.13 ab	1.03 ± 0.06 a
TVBN (mg/100 g)	15.96 ± 0.74 a	11.48 ± 0.28 b	9.80 ± 0.28 c

Different letters (a–c) in the same row indicate significant differences (*p* < 0.05).

## Data Availability

Data are contained within the article. The raw proteomics data have been uploaded to the iProX database (https://www.iprox.cn/), and its project ID is IPX0007849000.

## Supplementary Materials

The following supporting information can be downloaded at: https://www.mdpi.com/article/10.3390/foods13040525/s1. Figure S1: Ribosome pathway diagram; Figure S2: Glycine, serine, and threonine metabolism pathway map; Figure S3: Proteasome pathway diagram; Table S1: The DAPs identified with label-free analysis found in the SS vs. NT comparison; Table S2: The DAPs identified using 4D label-free analysis found in the UAS vs. NT comparison.
