# Supplementary material for: Effects of Quality Enhancement of Frozen Tuna Fillets Using Ultrasound-Assisted Salting: Physicochemical Properties, Histology, and Proteomics
Source: Foods. 2024 Feb 8;13(4):525. doi: 10.3390/foods13040525 (PMC10887591; doi:10.3390/foods13040525)
Supplement: Supplementary file 1 [file foods-13-00525-s001.zip › foods-2826086-supplementary.pdf]

Table S1 The DAPs identified by 4D label-free analysis found in SS vs NT comparison.
Table S2 The DAPs identified by 4D label-free analysis found in UAS vs NT comparison.

Figure. S1 Ribosome pathway diagram.
Figure. S2 Glycine, serine and threonine metabolism pathway map.
Figure. S3 Preteasome pathway diagram

Table S1. The DAPs identified by 4D label-free analysis found in SS vs NT comparison.

Accession	Description	Fold Change	Regulate
A0A0D6HEX0	30S ribosomal protein S2	32.00	up
A0A1A8UYI2	Carboxypeptidase Z	32.00	up
A0A6A5E9L6	Ryanodine receptor 3	32.00	up
A0A2K5RYN0	Neuropathy target esterase isoform X1	32.00	up
A0A6J2PA96	Titin-like	11.46	up
A0A673C6F1	Hematopoietic progenitor cell antigen CD34-like	2.17	up
A0A4W6EL06	Dihydropyrimidinase-related protein 3	2.05	up
A0A3Q1BF01	Catenin delta 2	2.01	up
O75369	Filamin-B	1.97	up
Q9C0E8	Endoplasmic reticulum junction formation protein lunapark	1.80	up
A0A8B9X473	Tubulin beta chain	1.80	up
A0A4W6BZ44	Fibrillin-1	1.77	up
A0A8C9ZW02	Myosin regulatory light polypeptide 9	1.66	up
A0A672YR28	Cellular retinoic acid-binding protein 2-like	1.53	up
A0A6G1QPP8	Cytochrome c oxidase subunit	1.49	up
A0A2I3BRX8	Muscleblind like splicing factor 2	1.47	up
A0A4U5UCU2	Neuroblast differentiation-associated protein AHNAK	1.46	up
Q14315	Filamin-C	1.42	up
A0A6P7HMI4	Nesprin-2	1.40	up
A0A7N8WWZ3	Aconitate hydratase, mitochondrial	1.40	up
I3JRM6	Copine-9	1.39	up
A0A8C4DAC4	Spectrin beta chain	1.32	up
A0A3Q4I6E8	proton-translocating NAD(P)(+) transhydrogenase	1.31	up
A0A4Z2BDY8	ATP-synt_ab_N domain-containing protein	1.30	up
A0A3Q1FAT4	Ribosomal protein L19	0.76	down
A0A2Y9JWH0	E2 ubiquitin-conjugating enzyme	0.76	down
A0A4W6E0V5	Complement C3-like	0.75	down
A0A2R8Y5P7	Histidine--tRNA ligase	0.75	down
W5PVR4	40S ribosomal protein S17	0.75	down
A0A668SG10	Ribosomal protein L5	0.75	down
A0A671YIZ3	60S ribosomal protein L27a	0.74	down
A0A668AZV5	Apolipoprotein M	0.73	down
G3QSE8	SMYD2 protein, partial	0.73	down
A0A8P4KRQ9	Alpha-2-macroglobulin	0.73	down
A0A8P4G730	Alpha-2-macroglobulin	0.73	down
A0A3P8TMU5	Protein argonaute-2	0.72	down
A0A6G1PYJ1	Peptidyl-prolyl cis-trans isomerase	0.72	down
A0A4W6C231	60S ribosomal protein L27a	0.72	down
P60901	Proteasome subunit alpha type-6	0.71	down

A0A2U9CGM8	AIG1 domain containing protein	0.70	down
A0A8J6ADN6	Endophilin-B1	0.70	down
A0A3Q1JTP2	60S ribosomal protein L13a	0.69	down
A0A8T2PP26	Proteasome subunit alpha type	0.69	down
A0A6G0I273	Scavenger receptor cysteine-rich type 1 protein M130 Soluble CD163	0.68	down
A0A7J8A684	Eukaryotic translation initiation factor 4A2	0.68	down
A0A5E4CK54	Casein kinase II subunit alpha	0.67	down
A0A665W3I9	Unc-45 myosin chaperone B	0.66	down
A0A4W6EAC0	Zinc binding alcohol dehydrogenase domain containing 2	0.66	down
A0A7N6FDT0	40S ribosomal protein S11	0.65	down
A0A673AGG9	Exportin-1	0.65	down
A0A8E0RL11	Ribosomal protein L3	0.64	down
A0A3P8T7T6	Myosin, heavy chain 10, non-muscle	0.63	down
I3MFT1	Proteasome subunit alpha type	0.63	down
P17252	Protein kinase C alpha type	0.62	down
A0A6J2RTN1	60S ribosomal protein L21	0.62	down
A0A7J7WEF8	40S ribosomal protein SA	0.61	down
P04040	Catalase	0.56	down
A0A157SWJ3	Glycerol-3-phosphate-binding periplasmic protein	0.54	down
A0A3P8RVW1	Betaine-homocysteine methyltransferase	0.54	down
A0A669F0Z4	Adenylosuccinate synthetase	0.53	down
A0A8C4DNK1	Tripartite motif-containing protein 54	0.52	down
A0A8C9Z2W3	Tryptase-2-like isoform X1	0.45	down
A0A3P9C4E7	GTPase IMAP family member 7-like	0.43	down
P46940	Ras GTPase-activati ng-like protein IQGAP1	0.43	down
L7N1C4	Tubulin beta chain	0.43	down
A0A6G0I169	Ribosome-releasing factor 2, mitochondrial	0.40	down
A0A6G0HWB5	Tryptase alpha/beta-1	0.37	down
A0A0D6GJX7	Tryptophan synthase beta chain	0.36	down
A0A254NFT7	Acetyltransferase component of pyruvate dehydrogenase complex	0.32	down
A0A0D6IWH2	50S ribosomal protein L1	0.30	down
A0A0D6IWT2	50S ribosomal protein L5	0.28	down
A0A672YCH6	Troponin I, fast skeletal muscle-like	0.23	down
A0A2K6UC87	Vacuolar proton pump subunit B	0.00	down
A9IIZ1	30S ribosomal protein S3	0.00	down
A0A254N3E4	DNA-directed RNA polymerase subunit beta	0.00	down
A0A8P4KPK4	Fibronectin type-III domain-containing protein	0.00	down

Table S2. The DAPs identified by 4D label-free analysis found in UAS vs NT comparison.

Accession	Description	Fold Change	Regulate
A0A1A8UYI2	Carboxypeptidase Z	32.00	up
A0A484C9N3	Ryanodine receptor 3	32.00	up
A0A2K5RYN0	neuropathy target esterase isoform X1	32.00	up
A0A0D6HEX0	30S ribosomal protein S2	32.00	up
A0A2S2Q4N0	T-complex protein 1 subunit epsilon	1.66	up
A0A813QT15	T-complex protein 1 subunit alpha	1.53	up
Q6ZWT7	Lysophospholipid acyltransferase 2	1.51	up
A0A6J3JCF0	COP9 signalosome complex subunit 5 isoform X1	1.37	up
A0A6I9PSR5	Calpain small subunit 1-like	1.34	up
A0A671V9Z8	AHNAK nucleoprotein	1.31	up
A0A7J8A684	Eukaryotic translation initiation factor 4A2	0.77	down
A0A4W6G1F7	Glucose-6-phosphate isomerase	0.77	down
A0A6G0HLR0	Microtubule-associated protein	0.75	down
A0A665WMJ5	60S ribosomal protein L9	0.74	down
A0A665WMJ9	Heat shock protein HSP 90-alpha	0.74	down
A0A8C4DHT3	Betaine-homocysteine methyltransferase	0.72	down
A0A8C9WUM5	coproporphyrinogen oxidase	0.72	down
A0A671YIZ3	60S ribosomal protein L27a	0.72	down
A0A8I3PLT0	60S ribosomal protein L13a	0.71	down
A0A3Q1HWE8	AP-2 complex subunit alpha	0.71	down
P02205	Myoglobin	0.70	down
A0A8X8BQQ2	Unc-45 myosin chaperone B	0.70	down
P07954	Fumarate hydratase, mitochondrial	0.69	down
A0A9J7EMQ5	ADP,ATP carrier protein （ADP/ATP translocase）	0.69	down
A0A3P8RVW1	Betaine-homocysteine methyltransferase	0.63	down
A0A8Q3WLN4	LIM zinc finger domain containing 1	0.61	down
A0A8C9ZW51	RAS like proto-oncogene A	0.59	down
A0A3Q3EKR2	Protein-glutamine gamma-glutamyltransferase 2-like	0.57	down
L7N1C4	Tubulin beta chain	0.56	down
P62890	Large ribosomal subunit protein eL30	0.56	down
P17252	Protein kinase C alpha type	0.56	down
A0A8C4I4V8	Adenylosuccinate synthetase	0.56	down
A0A8E0RL11	Ribosomal protein L3	0.55	down
A0A484CKC6	peptidylprolyl isomerase	0.55	down
A0A4W5LP36	Myosin tail domain-containing protein	0.53	down
A0A3P8S9R6	SEC22 homolog B, vesicle trafficking protein b	0.49	down
A0A254NCS3	ATP synthase subunit beta	0.49	down
A0A3P8T7T6	Myosin heavy chain 10	0.46	down
A0A3Q3X6Z4	Apolipoprotein M	0.44	down
A0A8C4DNK1	Tripartite motif containing 101	0.43	down
A0A1I6KL50	Elongation factor G	0.39	down

A0A672YCH6	Troponin I, fast skeletal muscle-like	0.38	down
A0A0D6H042	Putrescine-binding periplasmic protein	0.36	down
A0A2I4AWM6	Myosin-7-like	0.34	down
A0A0D6IWT2	50S ribosomal protein L5	0.32	down
A0A3Q1HUJ9	peptidylprolyl isomerase	0.31	down
A0A0D6GJX7	Tryptophan synthase beta chain	0.29	down
Q79GC6	Elongation factor Tu	0.26	down
A0A6G0I169	Ribosome-releasing factor 2, mitochondrial	0.21	down
A0A0D6IWH2	50S ribosomal protein L1	0.17	down
O66206	Chaperonin GroEL	0.07	down
A0A1I6LI20	Acetyltransferase component of pyruvate dehydrogenase complex	0.07	down
A9IIZ1	Small ribosomal subunit protein uS3	0.00	down
P46940	Ras GTPase-activating-like protein IQGAP1	0.00	down
A0A254N3E4	DNA-directed RNA polymerase subunit beta	0.00	down
A0A0D6GQ54	Outer membrane protein assembly factor BamD	0.00	down

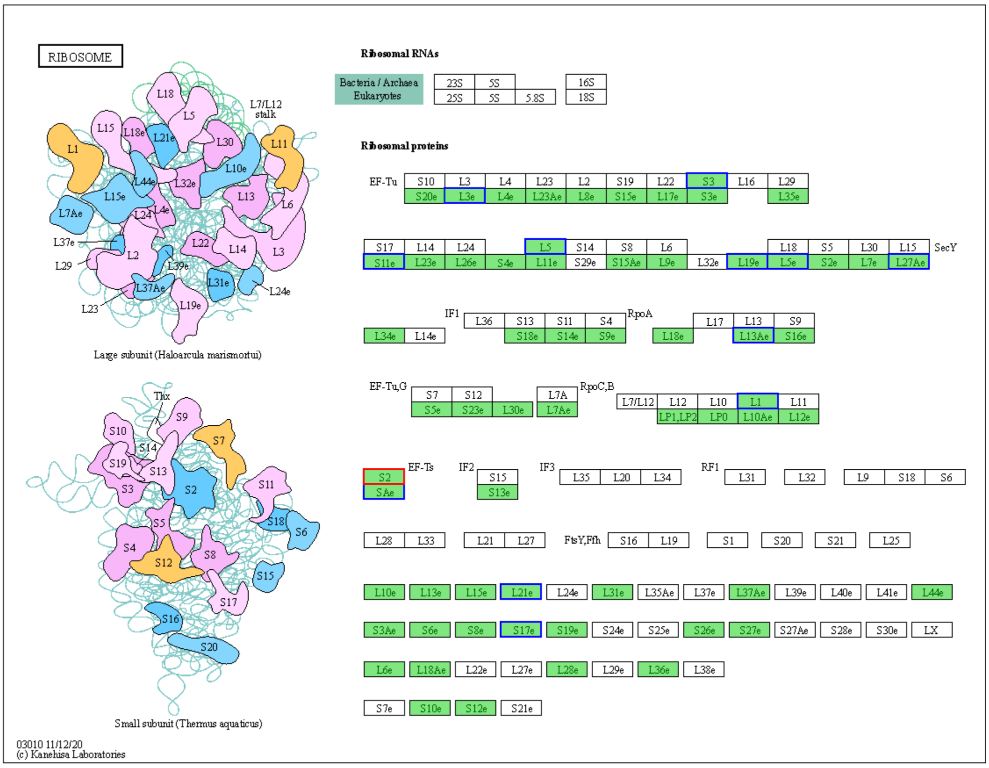

Figure S1. Ribosome pathway diagram. The green fills indicate the identified proteins in SS vs NT comparison, where red boxes indicate up-regulated DAPs and blue boxes indicate down-regulated DAPs.

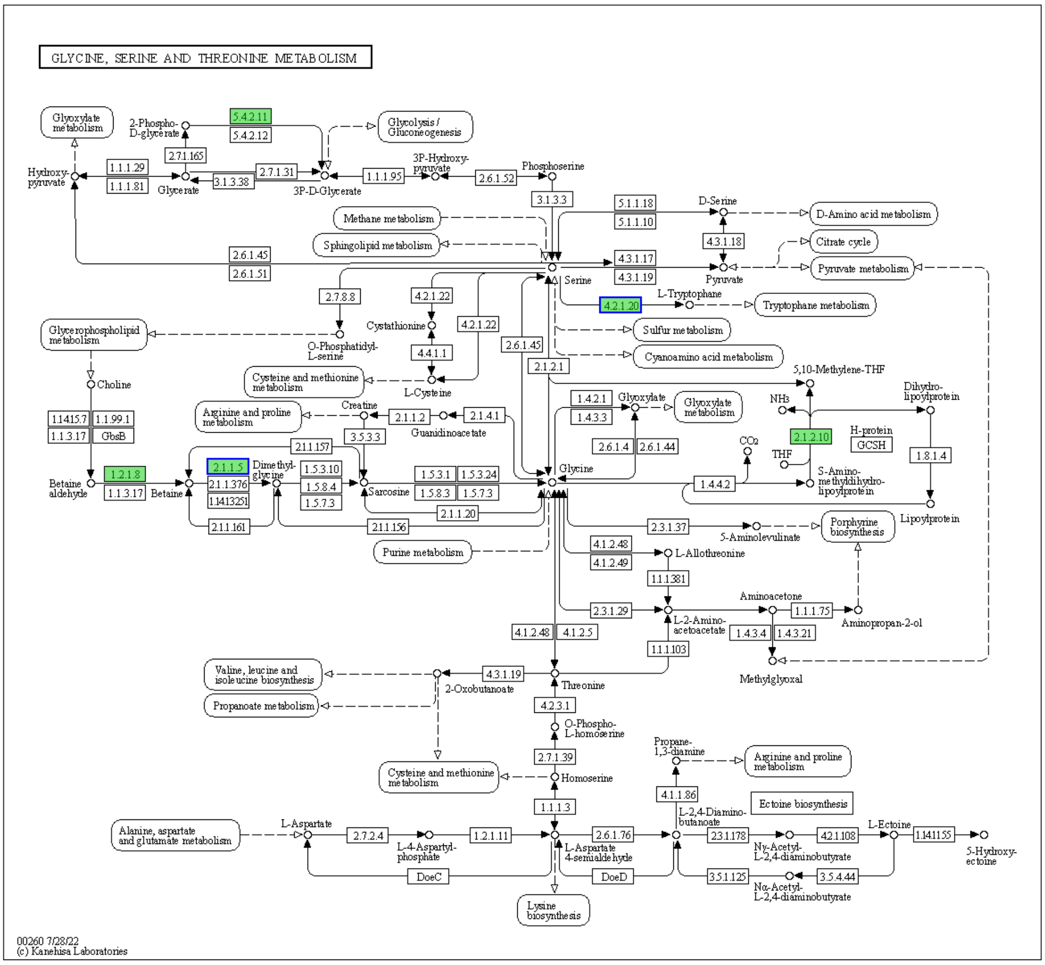

Figure. S2. Glycine, serine and threonine metabolism pathway map. The green fills indicate the identified proteins in UAS vs NT comparison, where red boxes indicate up-regulated DAPs and blue boxes indicate down-regulated DAP.

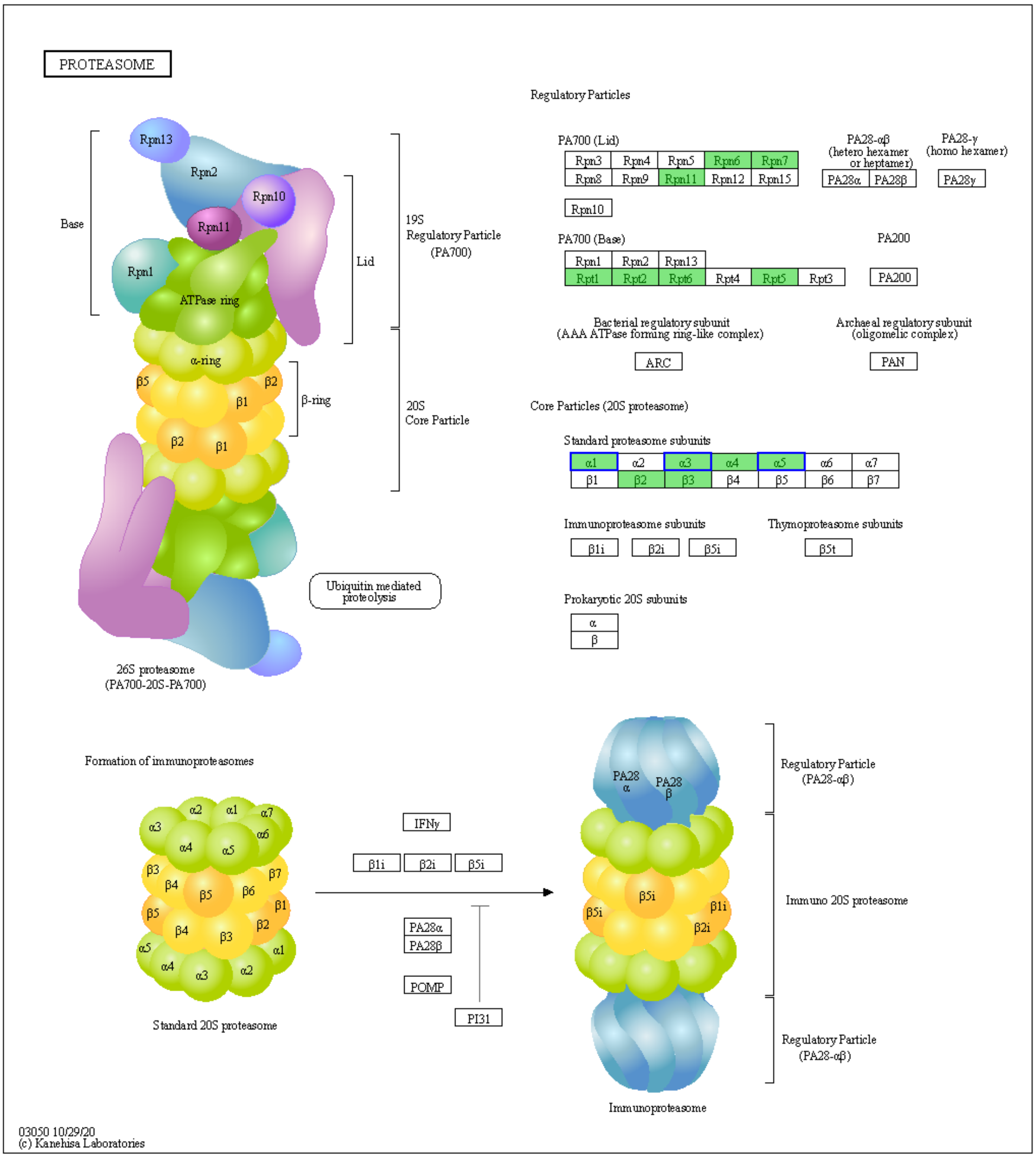

Figure. S3. Proteasome pathway diagram. The green fills indicate the identified proteins in SS vs NT comparison, where red boxes indicate up-regulated DAPs and blue boxes indicate down-regulated DAPs.
